# Combined Toxicity Effects and Health Risks of Microplastics and Multiple Pollutants

**DOI:** 10.1155/jt/3404011

**Published:** 2026-06-10

**Authors:** Lizhi Wu, Yuting Zhou, Mingluan Xing, Huixia Niu, Xueqing Li, Zhijian Chen, Xiaoming Lou

**Affiliations:** ^1^ Zhejiang Provincial Center for Disease Control and Prevention, Hangzhou, China, cdc.zj.cn; ^2^ School of Public Health and Emergency Management, School of Medicine, Southern University of Science and Technology, Shenzhen, China, sustc.edu.cn; ^3^ Center for Disease Control and Prevention of Jinyun County, Lishui, China

**Keywords:** combined toxicity, environmental health, microplastics, pollutants

## Abstract

Microplastics, as emerging pollutants, are ubiquitously present globally and pose significant threats to both ecosystems and human health. These pollutants not only exert direct toxicity on the environment and living organisms but also influence the toxicity of other pollutants through mechanisms such as adsorption and carrier effects. The health risks of microplastics are multifaceted and complex, involving cellular toxicity, immune dysfunction, and impairments to neurological, endocrine, and reproductive systems. This review summarizes the combined toxic effects of microplastics and heavy metals, organic pollutants, plastic additives, antibiotics, and viruses, exploring their potential mechanisms and influencing factors. The paper suggests that future research should focus on simulating exposure scenarios, examining the long‐term effects of exposure, and understanding the health consequences of combined exposures. This will provide new perspectives on the toxic effects of microplastics on the environment and organisms, enhance public awareness of their potential harm, and better address the impact of microplastic pollution on ecosystems.

## 1. Introduction

Plastics play a pivotal role in daily life and industrial production, and plastic pollution has emerged as a severe public health concern. According to data from the United Nations, the total weight of plastic produced globally each year is over 400 million metric tons, which is equivalent to approximately 2000 garbage trucks fully loaded with plastic waste being dumped into rivers, lakes, and oceans each day [1]. The increasing consumption of plastics, coupled with their persistence, has led to greater human exposure to microplastics (MPs). These particles are ubiquitous in food, drinking water, and even the atmosphere [2]. As an emerging environmental pollutant, MPs pollution has garnered significant attention on the international stage, with the UN plastic pollution treaty emphasizing the importance of addressing the hazards posed by MPs [3]. Recent researches have demonstrated that MPs not only have direct negative impacts on the environment and living organisms but may also interact with other pollutants or viruses to produce “combined toxicity effects,” amplifying their threat to ecosystems and public health. Therefore, understanding the key interactions and potential risks between MPs, pollutants, and viruses is of paramount scientific importance.

This review integrates interdisciplinary evidence, drawing from primary biological, microbiological, plant, and animal experiments. It clarifies the interactions between pollutants and MPs across nearly 100 studies, providing theoretical support for environmental health strategies based on mixed exposures. Such research is urgently relevant for achieving global sustainable development goals.

## 2. Basic Characteristics and Environmental Behavior of MPs

### 2.1. Definition and Classification of MPs

MPs were first introduced in 2004 by Thompson et al. from the Plymouth University in the United Kingdom, referring to plastic fragments and particles with a diameter of less than 5 mm [4]. Derived from the formation process, MPs can be divided into two types: primary and secondary. Primary MPs are directly generated by industrial processes as plastic pellets, such as those used in skincare products, toothpastes, and sunscreens, which include a significant number of primary MPs. Secondary MPs are plastic particles resulting from the physical, chemical, and biological breakdown of larger plastic debris, commonly found in the marine, river, and soil environments [5].

Based on size, “MPs” can be subdivided into nanoplastics (NPs) and MPs. NPs have a diameter of less than 1 μm, while MPs are those ranging in size from 1 μm to 5 mm. According to the chemical composition, they can be further categorized into polyvinyl chloride (PVC) MPs, polyethylene (PE) MPs, polypropylene (PP) MPs, polystyrene (PS) MPs, and polyamide (PA) MPs [6], with PS MPs being the most studied in terms of toxicity. MPs can also be classified by morphology into 5 primary categories: pellets, fibers, foams, fragments, and films [7]. According to their distribution, MPs can be roughly divided into marine, freshwater, and soil MPs. MPs of varying categories, due to differences in the chemical structure, surface properties, and solubility, exhibit diverse biological toxic effects.

### 2.2. Distribution Characteristics of MPs

Environmental science research indicates that these tiny plastic particles are not only distributed in traditional aquatic ecosystems, such as coastlines [8], rivers [9], and lakes [10], but also penetrated into remote areas such as deep‐sea environments [11], polar sea ice [12], and even high‐altitude snowfields [13], showcasing their strong environmental mobility. In terrestrial systems, agricultural soils [14] and sewage sludge from wastewater treatment plants [15] have become significant accumulation sites for MPs, while atmospheric deposition [16, 17] serves as a key pathway for their transboundary transport.

MPs have led to widespread exposure across the biosphere. From aquatic organisms, such as fish [18] and mussels [19], to terrestrial organisms, such as amphibians [20] and plants [21], MPs have accumulated in organisms across different trophic levels. Of particular concern is the exposure via food items, such as honey and table salt [22], and daily consumer products, such as cosmetics and synthetic textiles, which have inevitably brought MPs into human life. Furthermore, MPs have been shown to cross multiple biological barriers in the human body. They have been detected in major physiological systems such as the circulatory system [23], respiratory system [24], and digestive system [25], as well as in specialized organs with protective barriers such as the placenta [26] and testes [27], confirming their ability to penetrate tissues. The distribution of MPs across different human tissues varies significantly, which may be highly associated with their physicochemical properties, exposure routes, and local microenvironment characteristics [28].

### 2.3. Human Exposure Pathways and Accumulation of MPs

MPs can enter organisms via multiple exposure means, including ingestion, inhalation, and dermal contact. Among these, dietary intake represents the predominant route of exposure on account of the pervasive presence in marine and freshwater ecosystems [29]. Free‐floating MPs in water are easily ingested by shellfish and fish, accumulating in their bodies, and passing through the food chain, potentially causing illness or death in these organisms. Similarly, research by Gong Jiashun and others has shown a correlation between the substantial quantities of MPs in agricultural soils and in crops [30]. MPs in soil and organic fertilizers are likely absorbed by crops. Humans, at the top of the food chain, accumulate large amounts of MPs in their bodies through bioaccumulation, which poses unpredictable risks. Additionally, plastic fragments from ubiquitous daily plastic packaging can migrate into food, including table salt, honey, beer, and milk, posing a threat to food safety. Furthermore, MPs can be released into the air from synthetic textiles, dust, and tire wear, leading to respiratory exposure through inhalation. Cosmetic and skincare products and synthetic textile clothing may also cause MPs particles that enter the human body through skin exposure.

Upon entry into the human body, MPs do not fully excrete and instead accumulate. These persistent particles can harm health through mechanisms such as oxidative stress, inflammatory responses, immune dysfunction, metabolic disruptions, blocked cell proliferation, disruption of microbial metabolic pathways, organ development abnormalities, and carcinogenesis [31]. MPs can induce or contribute to cancer, gastrointestinal, pulmonary, cardiovascular, infectious, and inflammatory diseases [32]. Data of the potential adverse effects of MPs to various human organs and tissues continue to be updated. In April 2024, a study showed that MPs induced oxidative stress and inhibited antioxidant‐related protein activity, leading to skin and hair follicle damage in mice models, resulting in hair loss [33]. In June 2024, a study revealed for the first time that multiple MPs contaminated the endometrium in infertile women, pointing to factors within the uterine cavity caused by MPs exposure that led to decreased fertility [34]. In the same month, a research team discovered in a 3‐year study that individuals with MPs particles trapped in key blood vessels were more likely to develop heart disease, stroke, or die [35].

In conclusion, MPs pose significant potential threats to human health and the ecological environment, warranting attention and systematic research. Assessing the health impacts of MPs is of paramount importance.

## 3. Combined Toxicity Effects of MPs

In production and daily life environments, humans are often simultaneously or sequentially exposed to different chemicals from various environmental media, and the biological effects resulting from the combined action of multiple exogenous chemicals on the body are highly complex. In toxicology, the toxicity induced by simultaneous or sequential exposure to two or more chemicals is defined as a combined effect. These combined effects of multiple exogenous chemicals may occur simultaneously in living organisms and the external environment [36]. The types of combined effects are divided into noninteractive effects, such as additive or independent effects, and interactive effects, such as synergistic or antagonistic effects.

The pervasive utilization of plastics and the escalating prevalence of other environmental pollutants indicate an increasing likelihood of concurrent or sequential human exposure to MPs and such pollutants. This coexposure significantly increases the complexity of the resulting health effects. The unique physicochemical properties of MPs enable them to adsorb environmental contaminants through six primary mechanisms: (i) hydrophobic interactions, (ii) pi–pi interactions, (iii) electrostatic forces, (iv) hydrogen bonding, (v) van der Waals forces, and (vi) pore filling [37]. These MP–pollutant complexes cannot be fully eliminated after introduced into the organism and accumulate continuously, thereby inducing combined toxicity effects.

**FIGURE 1:**
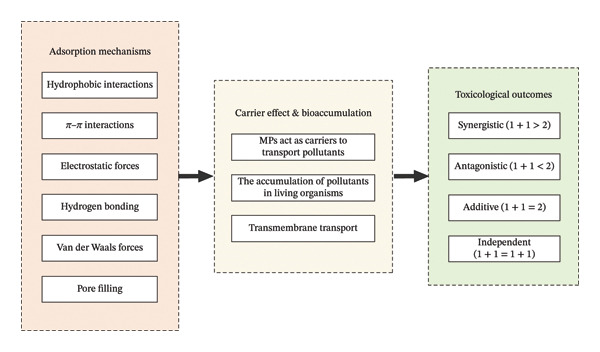
Interaction mechanisms between MPs and co‐existing pollutants. (Left) MPs adsorb environmental pollutants through multiple physicochemical mechanisms. (Middle) Once adsorbed, MPs act as carriers, facilitating pollutant transport and bioaccumulation in organisms. (Right) Combined exposure may result in different toxicological outcomes.

MPs and NPs have significant impacts in the environment as pollutant carriers [38, 39]. These plastic particles can accumulate various contaminants, such as persistent organic pollutants, plastic additives, heavy metals, and pharmaceutical residues, through surface adsorption. This carrier function substantially alters the bioavailability and environmental behavior of pollutants, enabling pollutants that would otherwise be degraded or fixed to re‐enter the biogeochemical cycle [40, 41]. The carrier impact of MPs is highly associated with the physicochemical properties. Parameters including particle size, specific surface area, surface charge, and degree of aging collectively determine their ability and selectivity to adsorb pollutants. Environmental factors, including ionic strength, pH, and temperature, also exert significant influences on this process [42, 43]. Notably, in real‐world environmental scenarios, MPs often coexist with a variety of pollutants, and interactions among these substances may result in complex effects, either enhancing or mitigating toxicity (Figure 1).

**FIGURE 2:**
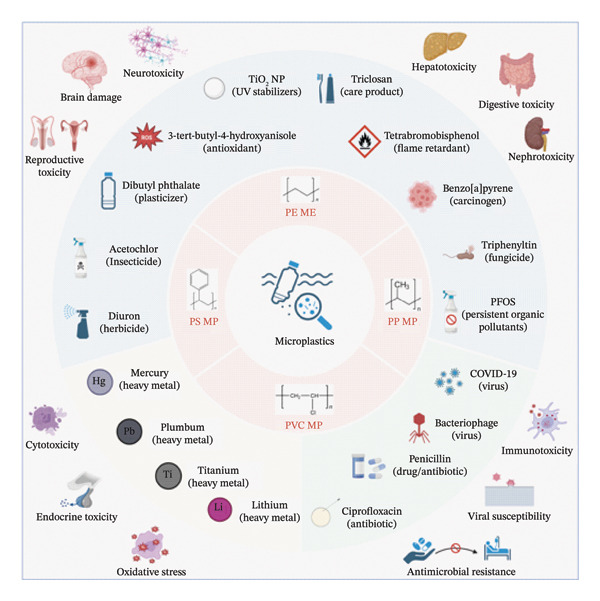
Combined toxicity effects and health risks of MPs and multiple pollutants. This schematic illustrates the major exposure routes (ingestion, inhalation, and dermal contact), types of copollutants (heavy metals, organic pollutants, plastic additives, antibiotics, and viruses), and associated health outcomes (cytotoxicity, neurotoxicity, reproductive toxicity, immunotoxicity, endocrine disruption, and oxidative stress) resulting from MPs coexposure.

A primary challenge in current risk assessment is the complexity of environmental exposure. Humans may encounter MPs–pollutant complexes through various routes, including dietary intake, inhalation, and dermal contact. Different exposure routes may result in significantly different toxicokinetic profiles. Additionally, variations in susceptibility across different life stages, the long‐term cumulative effects, and differences in individual susceptibility all contribute to the uncertainty in health risk evaluation [44]. These complexities underscore the importance of conducting combined exposure research based on real environmental scenarios (Figure 2).

## 4. Mechanisms of Combined Toxicity Effects of MPs and Multiple Pollutants and Associated Health Risks

### 4.1. Combined Toxicity Effects of MPs and Heavy Metals

Heavy metals encompass both toxic metallic elements including cadmium (Cd), lead (Pb), mercury (Hg), and arsenic (As), as well as essential trace metals including copper (Cu), magnesium (Mg), chromium (Cr), cobalt (Co), nickel (Ni), selenium (Se), tungsten (W), vanadium (V), manganese (Mn), molybdenum (Mo), iron (Fe), and zinc (Zn) [45]. These elements originate from diverse sources and are ubiquitously present in environmental media [46]. Exposure to heavy metals has become increasingly common due to industrialization and human activities. Sources of exposure may include industrial emissions, agricultural practices, fossil fuel combustion, waste disposal, and other human activities. Factors such as smoking and contact with commonly used commercial products, including paints, gasoline, electronics, pipes, and certain foods, can significantly increase the overall risk [47, 48].

Heavy metals are one of the most common pollutants, capable of damaging multiple organisms and being the main cause of occupational diseases. Heavy metal pollution remains a major challenge in the global environment, posing a serious threat to human life [49]. Soil contamination with heavy metals is particularly concerning, with excessive concentrations in crops raising health concerns regarding food safety. Current study between MPs and heavy metal mainly focuses on marine and agricultural ecosystems, with cadmium being the most frequently studied heavy metal and plants/rice as the primary model organisms.

Existing studies have shown that coexposure to MPs and heavy metals can exhibit antagonistic, synergistic, or additive effects [50], with the specific mode of action depending on the particle size, concentration, and surface properties of MPs, as well as the type of heavy metal and the species involved (Table 1). Existing empirical research has largely confirmed the presence of synergistic effects. For example, the coexposure to Cd and PS NPs enhanced the accumulation of Cd in the roots of *Arabidopsis thaliana* and aggravated oxidative stress [53]. Similarly, coexposure to PS NPs and CdCl_2_ in mice increased myocardial damage through the regulation of genes related to cell necrosis, apoptosis, and necroptosis [55]. Additionally, nanoscale NPs often exhibit stronger synergistic toxicity compared to micrometer‐scale MPs, as evidenced by the combined toxicity of PS NPs and silver nanoparticles (AgNPs) on ciliated protozoa, which was more severe than that of PS MPs alone [51].

**TABLE 1:** Summary of studies on the combined effects of MPs and heavy metals.

Class	Study subject	MPs/NPs	Chemical	Toxicity	Reference
Protist experiments	*Uronema marinum*	PS NPs	AgNPs	• Compared with individual exposure, the combined toxic effect of NPs and AgNPs on ciliates is enhanced. • The negative effect under the exposure of AgNPs and NPs is more pronounced than that of AgNPs and MPs. • Coexposure can interfere with the energy and lipid metabolism of cilia, thereby resulting in damage to DNA and proteins.	[51]
	*Euplotes vannus*	PS MPs/NPs	Cd	• The coexposure significantly elevated the level of reactive oxygen species (ROS) in ciliates and concurrently impaired the activity of antioxidant enzymes, thereby enhancing the oxidative damage in ciliates. • In contrast to MPs, the coexposure of Cd and NPs demonstrated higher negative influences.	[52]

Plant experiments	*Arabidopsis*	PS NPs	Cd	• The existence of PS NPs promoted the accumulation of Cd in the underground *Arabidopsis thaliana* seedlings, resulting in higher oxidative stress; compared with individual stress. • The combined stress had a more obvious influence on Arabidopsis thaliana. Higher concentrations of PS NPs impeded the migration of Cd from the roots to the leaves.	[53]
	*Oryza sativa* L.	PP MPs	Cd	• The independent exposure of PP, MPs, and Cd individually suppressed most germination parameters in *rice* seedlings; however, the coexistence (PP + Cd) mitigated the toxicity to a certain extent. • 13 μm PP + Cd exhibited an antagonistic effect on the growth of rice seedlings, while 6.5 μm PP + Cd showed a synergistic effect.	[54]

Animal Experiments	Male mice	PS NPs	CdCl_2_	• The coexposure influenced genes and proteins associated with apoptosis, necroptosis, and pyroptosis, leading to growth restriction and myocardial microstructural damage in mice and intensifying the toxic injury. • Exposure to PS NPs and/or Cd facilitated the ubiquitination of some proteins in mice myocardium.	[55]
	Zebrafish embryos	PS MPs/NPs	Hg	• The coexposure of PS MPs/PS NPs and Hg can restrain most of the toxic effects, and PS NPs are more effective than PS MPs in reducing the neurodevelopmental toxicity of Hg. The bioavailability of Hg is decreased, thereby alleviating the oxidative damage induced by Hg exposure in larvae.	[56]
	Planktonic copepods	MPs	Li	• The toxicological effects of Li and MPs exhibited antagonistic interactions at both the lowest and highest concentrations of the Li‐MPs mixture, while demonstrating synergistic interactions at intermediate concentrations.	[57]
	*Drosophila* larvae	PS NPs	Ag	• Minute plastic spots are capable of confining silver, preventing its bioavailability, and mitigating its potential impact.	[58]
	Zebrafish embryos	MPs	Cd	• MPs can potentiate the adverse effects of Cd on the early growth, oxidative damage and apoptosis of zebrafish.	[59]
	Mouse model	MPs	Pb	• The co‐exposure facilitated vascular smooth muscle cell loss, the release of inflammatory factors, and elastic fiber damage. • The coexposure promoted numerous ROS generation, disrupted mitochondrial function, and induced PANoptosome assembly and inflammation occurrence in vascular smooth muscle cell.	[60]
	Female mice	PS MPs	Pb	• The coexposure significantly augmented Pb accumulation in the ovaries, the histopathological impairments of the ovaries and uterus, the serum malondialdehyde level, as well as the decrease of serum superoxide dismutase and sex hormone levels. • The coexposure induced oxidative stress and activates the protein kinase R‐like endoplasmic reticulum kinase/eukaryotic initiation factor 2*α* (PERK/eIF2*α*) signaling pathway, thereby exacerbating ovarian toxicity.	[61]
	*Aquarana catesbeiana*	PE MPs	TiO_2_	• MPs decreased the survival rate and hatching rate of the group exposed to TiO_2_. • It might interact with the hatching enzymes of the embryos, hampering hatching and lowering their survival rate.	[62]
	*Tigriopus japonicus*	PS MPs/NPs	Hg	• MPs/NPs markedly increased Hg accumulation and augmented its toxic effect on *T. japonicus*. • NPs superposed on MPs exerted the maximal carrier effect on the Hg toxicity to *T. japonicus*, particularly in the incubation form.	[63]

Some studies have reported antagonistic effects. For example, coexposure to PS MPs/NPs and Hg reduced the bioavailability of Hg and its neurodevelopmental toxicity in zebrafish larvae [56]. Similarly, PP MPs exhibited antagonistic effects on *rice* seedling growth at specific particle sizes (13 μm) when exposed to Cd [54]. When MPs act as carriers of heavy metals, they may enhance bioavailability (e.g., PS NPs promote the accumulation of Hg in *Tigriopus japonicus* [63]). In contrast, the physical interactions of MPs with heavy metals, such as PS NPs binding Ag [58], or competition for binding sites may reduce toxicity. Additionally, concentration‐dependent effects are significant. For example, interactions between Li and MPs showed antagonistic effects at low/high concentrations, while at moderate concentrations, they exhibited synergistic effects [57]. Current data mainly focus on a few heavy metals including Hg, Pb, and Cd, and more studies are needed on other high‐risk environmental metals and real‐world multipollutant coexposure systems.

### 4.2. Combined Toxicity of MPs and Organic Pollutants

Organic compounds are a class of chemicals made up of carbon atoms, and they are widely present in industries, agriculture, and daily life. These compounds include pesticides, industrial chemicals, disinfection byproducts, personal care products, and more. The residue of organic pollutants in water, soil, and air has become increasingly problematic, especially in industrial and agricultural regions where concentrations often exceed safety standards. The toxicity of organic pollutants is diverse, including carcinogenicity, endocrine disruption, immune system suppression, and other effects. Prolonged exposure can lead to multiple health issues such as reproductive disorders, neurological damage, and chronic diseases [64].

The coexposure of MPs and organic pollutants is common in the environment. MPs not only adsorb organic contaminants but may also release harmful substances they carry during exposure. As a result, the simultaneous presence of MPs and organic contaminants presents organisms with a more complex toxicity risk. Current study between MPs and organic compounds mainly focuses on marine ecosystems. Common organic pollutants used in experimental designs include disinfection byproducts (DHP), herbicides (diuron), preservatives (parabens), pesticides (acetochlor), and personal care products (triclosan). Exposure subjects are mainly marine organisms such as medaka fish, zebrafish, and algae, with some studies using mouse models and cell lines. The combined toxicities studied are rich in diversity, including cytotoxicity, acute toxicity, reproductive toxicity, and neurotoxicity. By comparing exposure to MPs alone, organic pollutants alone, and combined exposure to both, these studies aim to determine whether the combined toxicity is independent, additive, antagonistic, or due to other interactions. Some experiments not only analyze the combined toxic effects using indicator data but also introduce variables such as exposure dose, exposure time, and MPs aging, which allow for a mechanistic analysis to explore the factors or mitigating elements affecting the combined toxicity of MPs and organic chemicals (Table 2).

**TABLE 2:** Summary of studies on the combined effects of MPs and organic compounds.

Class	Study subject	MPs/NPs	Chemical	Toxicity	Reference
Microbial Experiment	Freshwater diatoms and marine diatoms	PS MPs	Diuron	• The combined toxicity is antagonistic. • The adsorption behavior of MPs for diclofop reduced the cellular damage of diclofop to diatoms. • Atrazine reduced the physical damage of Microcystis to diatoms by alleviating oxidative stress.	[65]

Plant experiments	Dandelion seedlings	PS NPs	Dibutyl phthalate (DBP)	• The co‐exposure influences plant growth, induces oxidative stress, and alters the enzymatic and non‐enzymatic antioxidant levels of dandelions. • Carbohydrate, amino acid, and organic acid biosynthesis, along with energy metabolism, were all adversely affected.	[66]
	Soil‐wheat	PE NPs	Te‐germicide	• Synergistic toxicity affects the nutrient conditions and antioxidant mechanisms of the soil‐wheat system. • Joint exposure exacerbates oxidative stress in wheat and inhibits biomass.	[67]
	Multiple meadowsweet and sage roller blinds	PS MPs/NPs	2,2′,5,5′‐Tetrachlorobiphenyl (PCB‐52)	• The coexposure highly decreased the average specific growth rate and relative growth rate. • In comparison to the use of PS‐NPs alone, the coexposure to PS NPs and PCB‐52 exerted more ecological stress.	[68]

Animal Experiments	Male zebrafish	PS MPs/NPs	Tris(1,3‐dichloro‐2‐propyl) phosphate (TDCIPP)	• MNPs selectively promoted the bioaccumulation and distribution of TDCIPP, and NPs were more significant than MPs. • Emphasized the higher pollutant transfer potential of NPs in the context of combined exposure scenarios.	[69]
	*Caenorhabditis elegans*	PS NPs	Benzo[a]pyrene (BaP)	• BaP and PS significantly shorten the lifespan of *Caenorhabditis elegans* and lead to the occurrence of various aging phenotypes. • Coexposure to PS and BaP is related to the imbalance of glutathione homeostasis	[70]
	Adult zebrafish	PS MPs/NPs	Triphenyl phosphate (TPHP)	• The exposure of MPs and NPs had no important impact on the acute toxicity of TPhP, exacerbated the reproductive toxicity of TPHP. • The effect of PS on the reproductive toxicity of TPHP was gender‐dependent.	[71]
	Marine guppy	PS MPs/NPs	Triphenyltin (TPT)	• NPs + TPT (NT) is more toxic than MPs + TPT (MT). • MT is more likely to cause hormonal imbalances compared to NT. • The size of the particles determined importantly the severity of these effects.	[72]
	Zebrafish	PS NPs	2,4‐Di‐tert‐butylphenol (2,4‐DTBP)	• The effects of coexposed on male feeding ability and gut tissue pathology were enhanced, while those on female fish were attenuated. • Coexposure stimulated female fish to reshape their microbial composition, potentially enhancing exogenous biodegradation, while Aeromonas hydrophila aggravated inflammation in male fish.	[73]
	Male zebrafish	PS NPs	N‐(1,3‐Dimethylbutyl)‐N′‐phenyl‐*p*‐phenylenediamine‐quinone (6PPDq)	• Zebrafish exposed to 6PPDq exhibit obvious hyperactivity, and this effect is more pronounced after coexposure with PS‐NPs. • The molecular mechanisms are associated with genes related to neurotransmitters and fatty acid metabolism.	[74]
	Adult zebrafish	PS NPs	4‐Methylbenzylidene camphor (4‐MBC)	• The coexposure promoted the 4‐MBC load across all tested tissues. • The coexposure induced higher pathological damage to brain tissue and induced sex‐specific neurotoxicity and reproductive disruption than the 4‐MBC exposure alone. • Female zebrafish manifested autism‐spectrum‐disorder‐like behaviors and oocyte development disruption, while male zebrafish displayed Parkinson’s‐like behaviors and spermatogenesis disruption.	[75]
	*Platynereis dumerilii*	PS MPs/NPs	Dibutyl phthalate (DBP)	• When *P. dumerilii* were in the nonfeeding early stage, NPs significantly enhanced the toxicity of DBPs. • MPs did not interact with *P. dumerilii* embryos, while NPs strongly aggregated on the chorion of the embryos and completely wrapped the embryos.	[66]
	Zebrafish	PE MPs	Acetochlor (ACT)	• PE MPs importantly exacerbated the acute toxicity of ACT, promoted ACT accumulation in zebrafish, and aggravated oxidative stress damage in intestinal tissues. • Coexposure to PE MPs and ACT can cause mild tissue damage and changes in the composition of intestinal microbiota in zebrafish.	[76]
	Mouse testes	PS NPs	Lipopolysaccharide (LPS)	• PS‐NPs aggravated testicular dysfunction induced by LPS. • In mice exposed to combined exposure, the total sperm count in the testis, testosterone levels in both the blood and testis, and the expression level of StAR, an enzyme involved in steroidogenesis, were significantly lower.	[77]
	Female mice	PS MPs	3‐tert‐Butyl‐4‐hydroxyanisole (3‐BHA)	• The coexposure exacerbated female reproductive toxicity. • It increased the pathological abnormalities of the ovaries and uterus and reduced the levels of female sex hormones such as follicle‐stimulating hormone (FSH).	[78]
	Macrocarpus	PS MPs	Tri‐n‐butyl phosphate (TnBP)	• The PS MPs and TnBP have a synergistic effect on growth toxicity and intergenerational effects. • The TnBP mitigated oxidative stress by weakening the bioaccumulation of PS MPs.	[79]
	Xenopus tadpole	aPE MPs	Triclosan (TCS)	• The coexposure of aPE MPs and TCS exacerbates oxidative stress and neurotoxicity, leading to more intense oxidative stress and inflammation.	[80]

Current studies indicate that MPs can function as vectors for organic contaminants, enhancing toxicity by promoting bioaccumulation. For example, PS NPs significantly increase the distribution of organophosphorus flame retardant TDCIPP in zebrafish [69], while PE MPs increase antibiotics (ACT) accumulation in the gut and exacerbate oxidative damage [81]. This carrier effect is especially prominent in nanoscale MPs (NPs), as PS NPs more significantly promote ecological stress in plants by enhancing PCB‐52 accumulation [67]. Additionally, interactions between MPs and organic pollutants may interfere with the bioavailability of pollutants. For instance, PS MPs reduce the cell damage caused by diuron to diatoms [68], while NPs encapsulating DBP enhance its toxicity to *Platynereis dumerilii* [82]. Combined exposure toxicity also exhibits gender differences and tissue specificity. For example, coexposure to PS NPs and 2,4‐DTBP exacerbates intestinal inflammation in male zebrafish but enhances degradation capabilities in female fish by remodeling their gut microbiota [73]. PS NPs coexposure with 4‐MBC induces autism spectrum behavior and oocyte developmental disorders in female zebrafish, while male zebrafish show Parkinson‐like symptoms and abnormalities in spermatogenesis [75]. Some studies have revealed dose‐dependent effects. For example, PS MPs and TnBP display antagonistic effects at low concentrations by reducing MPs accumulation, but at high concentrations, they show synergistic growth toxicity [78], suggesting a nonlinear dose–response relationship in real environmental exposure scenarios. Future study could further investigate the molecular mechanisms between MPs and organic pollutants, focusing on the ecological risks of long‐term low‐dose exposure at the population level.

### 4.3. Combined Toxicity Effects of MPs and Plastic Additives

Plastic additives are chemicals added during the polymer processing to enhance their processing performance or to compensate for deficiencies in the resin itself [83]. Different types of additives are required to meet various processing and application needs. In recent years, emissions of plastic additives have increased dramatically, yet their pollution status has not received sufficient attention. Furthermore, there is a lack of systematic strategies for the effective management of plastic additive pollution. Therefore, analyzing the hazards and current contamination status of plastic additives and exploring efficient control strategies is crucial [84].

Currently, plastic additives are classified into nine categories: antioxidants, antistatic agents, foaming agents, flame retardants, lubricants, impact modifiers, plasticizers, colorants, and fillers [85]. Although these additives improve the performance of polymer products, many of these substances are toxic and have high risk of pollution in water, air, and soil [86, 87]. Due to their widespread production and use, MPs and their additives had been detected multiple times in aquatic ecosystems. These emerging pollutants may exhibit bioaccumulation and could potentially harm aquatic organisms at environmentally relevant concentrations [88]. Plastic additives exhibit developmental toxicity, reproductive toxicity [89], hepatotoxicity [90, 91], and neurotoxicity [92]. These additives can migrate and continuously expose humans through food‐contact materials. Therefore, it is critical to ensure that the emission and contamination of high‐priority substances are properly managed, safeguarding the human health and environment [85].

The current studies demonstrate that the combined toxicity of MPs and plastic additives shows significant concentration dependence and dynamic release characteristics (Table 3). For example, low concentrations of PS MPs and DBP show no significant toxicity to *Streptomyces azura M145*, but at high concentrations, they exhibit antagonistic effects [93]. The combined toxicity of PS MPs and APFO fluctuates between synergistic and antagonistic effects depending on the concentration ratio [97]. MPs’ physical interaction with additives may delay their toxic release. For example, PS MPs encapsulating TBBPA significantly delay the lethal effects on tadpoles [99], but long‐term exposure to additive slow‐release may lead to chronic cumulative toxicity. Coexposure of PE MPs and UV stabilizers inhibits frog embryo hatching by adhering to the embryo’s gelatinous capsule [62]. Additives may also reverse the environmental behavior of MPs, such as DBP promoting MP aggregation in marine copepods, thereby reducing their bioavailability [98]. These results suggest that the interaction between MPs and additives involves not only a simple carrier‐loading relationship but may also indirectly affect toxicity through changes in the aggregation state of MPs or biointerface characteristics.

**TABLE 3:** Summary of studies on the combined effects of MPs and plastic additives.

Class	Study subject	MPs/NPs	Chemical	Toxicity	Reference
Microbial experiment	*Streptomyces azura M145*	NPs	Dibutyl phthalate (DBP)	• When NPs and DBP are present at low concentrations, coaddition is nontoxic to M145. However, as the concentration increases and the combination concentration changes, coaddition produces joint toxicity to M145 and exhibits antagonistic effects. • The key reason for the difference in cellular toxicity is the change in NP agglomeration and DBP adsorption on NPs, and the size of this toxicity is influenced by the combination concentration.	[93]

Animal experiments	Mouse model	PS MPs	Di(2‐ethylhexyl) phthalate (DEHP)	• The coexposure of PS MPs and DEHP induces oxidative stress, activates the nuclear factor‐kappa B (NF‐κB)/NLRP3 pathway, and aggravates renal pyroptosis and inflammation. • The antioxidant N‐acetylcysteine (NAC) and the NLRP3 inflammasome inhibitor (MCC950) have been demonstrated to mitigate the changes induced by the aforementioned interventions.	[94]
	Zebrafish embryos	PS MPs	Bisphenol S (BPS) Mono(2‐ethylhexyl) phthalate (MEHP)	• At low concentrations, exposure to the mixture could lead to embryonic damage and developmental deformities in zebrafish embryos, specifically depending on the combination of mixture concentrations.	[95]
	Male mice	PS MPs/NPs	Phthalate esters (PAE)	• The coexposure of PAE and MPs induces an enhanced reproductive toxicity, manifested as more significant alterations in sperm physiology and spermatogenesis. • The increased reproductive toxicity is not only induced by PAE, but may also be related to the oxidative stress effect caused by MPs.	[96]
	Macrocarpus	PS MPs	Ammonium perfluorooctanoate (APFO)	• The combined toxicity pattern fluctuates between antagonism and synergy in accordance with the concentration ratio of MPs to APFO. • Joint toxicity takes place in the intestine of water fleas, within the physiological and biochemical reactions mediated by intestinal obstruction, and observable intestinal damage can be witnessed.	[97]
	Female mice	PS NPs	3‐tert‐Butyl‐4‐hydroxyanisole (3‐BHA)	• The coexposure increased the histopathological abnormalities of the ovaries and uterus, reduced FSH and LH, and simultaneously increased antioxidant activities of catalase (CAT) and glutathione peroxidase (GSH‐Px).	[78]
	Bullfrog embryos	PE MPs	Ultraviolet (UV)	• MP decreased the survival rate and hatching rate of the TiO_2_ groups. • It is possible to prevent hatching and reduce their survival rate. • Osmotic regulation might be affected by the adherence of MPs to the gelatinous capsule of the embryo.	[62]
	Marine copepods	PS MPs	Dibutyl phthalate (DBP)	• Antagonistic toxic effects between the two contaminants were observed in both acute and chronic tests. The former was due to the adsorption of MPs, which resulted in the decreased bioavailability of DBP, while the latter was attributed to the promotion of MPs aggregation in the presence of DBP.	[98]
	Xenopus tadpole	PS MPs	Tetrabromobisphenol A (TBBPA)	• The toxicity of sole exposure to TBBPA is the greatest, while the encapsulation of TBBPA in MPs importantly delays acute lethal toxicity towards tadpoles by reducing the rapid and substantial release of TBBPA. • Compared to TBBPA used alone, both pristine PS MPs and MPs containing 10% TBBPA demonstrated delayed survival toxicity.	[99]

Existing data mainly focus on plasticizers and flame retardants, and research should be expanded to include other high‐priority additives (e.g., perfluorinated compounds) and real environmental factors, including UV exposure and microbial decomposition. Furthermore, specific blocking strategies for combined MPs and additive pollution, such as the application of NOD‐like receptor family pyrin domain containing 3 (NLRP3) inhibitors (e.g., MCC950) [94], need to be developed to provide theoretical support for ecological and health risk management.

### 4.4. Combined Toxicity Effects of MPs and Antibiotics

Antibiotics are a class of secondary metabolites produced by microorganisms, such as bacteria, fungi, and actinomycetes, or by higher plants and animals. They exhibit antimicrobial activity against pathogenic microorganisms along with various other biological effects [100]. At low concentrations, antibiotics can inhibit or eliminate pathogenic microorganisms and interfere with the normal growth and metabolic processes of other living cells. [100]. These substances are usually classified into categories including β‐lactams, aminoglycosides, macrolides, tetracyclines, sulfonamides, glycopeptides, and quinolones, according to their mechanisms of action and chemical structures [101]. The discovery of antibiotics greatly advanced human healthcare, promoted disease prevention and treatment, and has expanded their application across various industries [102]. However, as global antibiotic consumption continues to increase, new antibiotic pollutants continue to appear in the environment, and the misuse of antibiotics has become a growing concern worldwide due to its profound negative impacts on public health and the environment [103].

Antibiotics primarily enter the environment from a variety of sources such as untreated domestic sewage [104], wastewater treatment plants [105], hospitals [106], pharmaceutical wastewater [107], livestock farming [108], aquaculture [109], and crop production [110]. Fluoroquinolones, macrolides, tetracyclines, and sulfonamides have become common pollutants in global environmental matrices [111]. Current research on the toxicity of environmental antibiotic contamination primarily focuses on the antibiotics themselves, with relatively little emphasis on their combined toxicity with other pollutants. Some researchers have begun studying the coexposure of antibiotics and MPs (Table 4).

**TABLE 4:** Summary of studies on the combined effects of MPs and antibiotics.

Class	Study subject	MPs/NPs	Chemical	Toxicity	Reference
Microbial experiment	*Antibiotic-resistant bacteria*	PS NPs	Clarithromycin (CLA)	• PS NPs can adsorb CLA, resulting in structural alterations of insulin and influencing its physiological functions. PS NPs decrease the inhibitory effect of CLA on pathogenic bacteria, consequently promoting antibiotic resistance. • There exist both synergistic and antagonistic effects between PS NPs and CLA.	[112]
	*Microcystis aeruginosa*	PS MPs	Chloramphenicol (CAP)	• Both individual and combined treatments pose a threat to algal growth, with combined application exhibiting higher toxicity. • This synergistic effect can be attributed to the similar targeting of toxicological impacts on photosynthesis by both CAP and MPs, as well as the oxidative stress induced by their interaction.	[113]
	*Microalgae chlamydomonas reinhardtii*	PS MPs	Sulfadiazine (SDZ)	• The individual and combined use of MPS and SDZ both inhibited the growth of microalgae, and the combined use decreased the inhibition rate. The adsorption of MPs could mitigate the combined effect of MPs and SDZ.	[114]

Plant experiments	Lettuce seedlings	PE MPs	Oxytetracycline (OTC)	• Joint exposure significantly promoted the Simpson index of the rhizosphere bacterial community and altered community composition. • Significantly changed the rhizosphere and nonrhizosphere metabolome profiles.	[115]

Animal experiments	*Corbicula fluminea*	PS MP	Ciprofloxacin (CIP)	• The existence of PS lowers the toxicity of CIP in the digestive glands. • The cotreatment group of NPs aggravates the siphon inhibition rate of C. fluminea.	[116]
	Adult zebrafish	PS MPs/NPs	Oxytetracycline (OTC)	• NPs, OTC, and their joint exposure result in intestinal epithelial injury, while co‐exposure with MPs reduces the intestinal damage caused by single OTC exposure.	[117]
	Mice	PS NPs	Aflatoxin B1 (AFB1)	• Colonic inflammation and intestinal barrier impairment are exacerbated, manifesting a more severe inflammatory response. • NPs aggravate AFB1‐induced intestinal microbiota dysregulation and fecal metabolome remodeling. Coexposure intensifies AFB1‐induced liver fibrosis and inflammation.	[118]
	Mussel	PS NPs	Norfloxacin (NOR)	• PS NPs will increase the effects of NOR on mussel antioxidant and immune defense capabilities. • The toxic effects under different concentrations of NOR show different expressions, and relevant factors, enzymes, and gene expression levels change accordingly.	[119]
	Zebrafish model	PS MPs	Oxytetracycline (OTC)	• Simultaneous exposure to microplastics and ofloxacin in zebrafish resulted in increased lipid accumulation in the liver, elevated levels of triglycerides and cholesterol, as well as more severe inflammatory responses and heightened oxidative stress. • The proportion of the microbiome composition in the intestinal contents undergoes alterations, with a significantly elevated level of endotoxin lipopolysaccharide (LPS) derived from intestinal bacteria, and reduces the gene expression and activity of lipase.	[120]
	Zebrafish embryos/larvae	PS MPs	Penicillin (PNC)	• The coexposure of nonteratogenic concentrations of the PS MPs and PNC synergistically inhibits the behaviors of embryonic heartbeats and spontaneous movements, touch responses, and larval swimming behavior responses. • Coexposed to PNC and PS MPs demonstrates antagonistic effects, and there exists a certain toxicity. • There are synergistic effects of aged PS MPs and PNC on apoptosis, *ROS* formation, and the regulation of neurotransmitter metabolites.	[121]
	Daphnia Magnia	PS MPs	Roxithromycin (ROX)	• Coexposure to different sizes of PS MPs significantly reduces the reaction of various biomarker enzymes. • Coexposure to *ROX* and 1 μm PS MPs leads to the most severe biological response.	[122]
	Zebrafish embryos	PS MPs	Sulfamethoxazole (SMZ)	• Coexposure at multiple areas and stages of zebrafish development induced mortality and deformities. • Coexposure also induced extensive endocrine toxicity in zebrafish. • Toxicological antagonism exists between PS MPs and SMZ, resulting in reduced combined toxicity.	[123]
	Mammalian intestines	PS MPs	Tetracycline (TCH)	• PS‐MPs mainly damage the colon, whereas TCH predominantly damages the small intestine, particularly the jejunum. • PS‐MPs and TCH affect the metabolic processes of the microbial community, especially protein absorption and digestion.	[124]
	Mice	PS MPs	Doxycycline (DOX)	• The exposure of DOX in combination with PS disrupted the homeostasis of the intestinal microbiota, subsequently inducing brain damage and inflammation via the gut‐brain axis and simultaneously led to the decline of learning and memory behaviors.	[125]
	Yellowfin sea bream	MPs	Florfenicol (FLO)	• Liver biomarkers are more sensitive to pollutants, and co‐exposure to FLO and MPs induces more pronounced and longer‐lasting toxicity.	[126]

Antibiotic‐resistant genes (ARGs) are genetic elements that provide bacteria with the ability to resist antibiotics. These genes encode mechanisms such as inactivating enzymes, efflux pumps, target‐modifying proteins, or membrane permeability regulators to reduce the effectiveness of antibiotics. ARGs are found in chromosomal DNA, plasmids, transposons, or integrons and can spread rapidly between bacterial strains through horizontal gene transfer (e.g., conjugation, transformation, and transduction), facilitating the spread of resistance [127, 128]. The evolution of ARGs is driven by antibiotic misuse, coselection of gene elements, and mutation accumulation, which accelerates the enrichment of resistance genes in both the environment and pathogens. The global spread of ARGs has led to the emergence of multidrug‐resistant and pan‐drug‐resistant bacteria, posing a severe risk to clinical infection treatment and public health. The cross‐transmission chain in the environment, such as between water, soil, and the “One Health” concept, further exacerbates the risk [129].

The rapid development of the medical industry, industrial production, soil cultivation, and aquaculture has caused the release of large amounts of wastewater containing novel contaminants such as antibiotics, MPs, and ARGs. These mixed pollutants are widespread. MPs, because of their high surface area, hydrophobicity, and heterogeneous charge, can efficiently adsorb bacteria carrying ARGs or free DNA fragments. In marine environments, MPs can transport ARGs across long distances through ocean currents, forming cross‐regional transmission networks. Furthermore, when MPs undergo aging through ultraviolet (UV) or microbial processes, their surface roughness increases, and oxygen‐containing functional groups form, further improving their ability to interact with resistant bacteria.

The MPs–microbe interface provides a unique microenvironment for bacterial gene exchange, promoting horizontal gene transfer [130]. Studies have shown that extracellular polymeric substances (EPS) adsorbed onto MPs can lower cell membrane permeability, facilitating the transfer of plasmids during conjugation. Coexposure to MPs with antibiotics, heavy metals, and other contaminants can produce synergistic selective pressure, and the increased chemical pollutants on MPs can prompt the emergence of resistance genes under the selective pressure from bacterial communities and horizontal gene transfer [131]. MPs can also accumulate antibiotics, ARGs, and microbes from their surroundings, with salinity increasing the reduction of antibiotic adsorption and ARG abundance on MPs [132].

The interaction between MPs and antibiotic resistance is a new environmental threat. We should pay attention to how the interaction between MPs and ARGs affects the ecological impact and potential harm to the environment. Preventing the misuse of plastic products and antibiotics in the environment, along with investing more research into understanding the interactions between these pollutants and the environment, is critical for establishing effective management systems to mitigate the associated risks.

### 4.5. MPs and Biological Contaminants

MPs can act as a solid scaffold for microorganisms, viruses, and various biological molecules such as lipopolysaccharides, allergens, and antibiotics, with these interactions largely dependent on the MPs’ size, chemical composition, and surface charge [133]. As potential vectors for viruses, MPs may extend viral survival time and enhance infectivity [134]. Li et al. identified 1719 distinct types of viruses associated with MPs, with the highest diversity of viruses found in PP, where approximately 250 specific viruses were detected [135].

There is evidence showing that MPs can interfere with the interactions between viruses and their hosts (Table 5). This direct interference is affected by a multitude of factors, such as the form of MPs, viruses, host species, host‐virus load, and immune responses. For instance, Deng et al. found that ingestion of PS MPs increased the susceptibility of bees to viral infections [136]; studies by Shan and Wang et al. demonstrated that MPs enhanced the susceptibility of shrimp to white spot syndrome virus (WSSV) by influencing both viral virulence and host immune defense, with PS MPs significantly enhancing the ability of the influenza virus to infect host cells [137, 142]. Wang et al. observed that PS NPs altered the replication of viruses in the spleen, brain tissue, and spleen cells of orange‐spotted grouper [138]. Seeley and Yan reported that MPs increased virus‐mediated fish mortality, with mortality rates correlating with host‐virus load, immune responses, and mild gill inflammation [139]. Ochirbat et al. found that the heterogeneity of virus particles and MPs reduced the quantity of active bacteriophages in aquatic environments, relying on the zeta potential of the polymer and the type of phage [140]. Khan et al. highlighted the potential effect of MPs nanoparticles (M‐NPLs) in increasing the latency period, spread, and transport of COVID‐19 viruses [143].

**TABLE 5:** Summary of studies on the combined effects of MPs and biological contaminants.

Class	Study subject	MPs/NPs	Chemical	Toxicity	Reference
Virus	Honeybee	PS MPs	Honeybee viruses	• Substantial quantities of ingested PS MPs accumulate within honeybees, thereby heightening their susceptibility to viral infections. • Following ingestion of PS MPs, significant alterations were observed in genes associated with membrane lipid metabolism, immune response, detoxification, and the respiratory system.	[136]
	Cell	PS MPs	Influenza A virus	• PS downregulates RIG‐I and inhibits TBK1 phosphorylation, thereby reducing IRF3 and its active form P‐IRF3. This significantly decreases IFN‐β expression and impairs the intracellular innate antiviral immune response.	[137]
	Orange‐spotted grouper	PS NPs	Fish virus	• Exposure to PS NPs results in downregulated expression levels of Toll‐like receptor genes and interferon‐related genes, along with a marked decline in the capacity to suppress viral replication in cells and tissues, both prior to and following viral infection.	[138]
	Fish	MPs	Unspecified fish virus	• When fish are simultaneously exposed to viruses and MPs, the mortality rate increases significantly. MPs may have significant impacts on population health when facing other stressors.	[139]
	Aquatic environment	MPs	Bacteriophages	• The adsorption mechanism can completely remove viral particles, while leachables (MPs additives) can inactivate up to 50% of phages.	[140]
	Aquatic environment	PVC MPs	MS2 bacteriophage	• Higher concentrations of PVC MPs result in lower ultraviolet transmittance, which subsequently reduced the inactivation rate of MS2 bacteriophage and hindering the disinfection process.	[141]

Additionally, MPs can indirectly affect virus populations by influencing environmental elements. For example, Hu et al. found that higher concentrations of PVC MPs reduced UV transparency, leading to a decrease in the inactivation of *MS2 bacteriophage*, potentially interfering with disinfection processes and affecting drinking water safety and treatment [141].

In summary, current studies on the combined effects of MPs and viruses focus on the following areas: (1) the interactions between viruses and MPs, especially changes in viral virulence; (2) the viral populations, diversity, and functionality within plastic spheres; (3) the impacts of viruses and MPs on host‐related environments, with data mainly focusing on host immune responses and mortality rates [144]. However, there is still limited molecular‐level mechanistic exploration, which warrants further investigation.

### 4.6. Acute Versus Chronic Exposure Effects of Combined Toxicity Effects

The combined toxicity effects of MPs and copollutants vary significantly between acute and chronic exposure scenarios [145]. Acute exposure typically involves short‐term, high‐dose exposure and triggers immediate physiological responses. For instance, acute exposure to PS MPs induced behavioral changes and inflammation in mice within 3 weeks [146], while zebrafish exposed to PE MPs exhibited metabolic disturbances and gut dysbiosis [147]. In pregnant rats, nano‐PS translocated to fetal tissues within 24 h postexposure [148]. Acute MP exposure has also been shown to impair hydrogen production in anaerobic granules [149], induce immune responses in silkworms, and disrupt ion regulation in zebrafish embryos [150].

In contrast, chronic exposure entails long‐term, low‐dose exposure, which may lead to cumulative toxicity and multiorgan damage. Mice exposed to PS MPs for 180 days showed MP accumulation in the brain, neuroinflammation, and cognitive deficits [151]. Chronic exposure also induced male reproductive toxicity and renal fibrosis through ferroptosis [152, 153]. In *Drosophila*, chronic PTFE MP exposure altered energy metabolism and sleep patterns [154]. Hepatic effects included liver injury and gut microbiota dysbiosis in mice [155], and increased chemosensitivity in human liver cells [156]. In marine medaka larvae, chronic low‐dose MP exposure caused intestinal inflammation [157].

Studies comparing acute and chronic effects further highlight these differences. While acute PS MPs exposure in *Bombyx mori* induced immune responses without affecting survival [158], chronic coexposure to cadmium and PS NPs induced oxidative stress and ferroptosis in mouse kidneys, effects more severe than those in acute models [159]. Acute studies identify immediate hazards, while chronic studies are essential for understanding long‐term health risks and cumulative damage.

### 4.7. Key Pathways and Mechanisms in MPs–Pollutants Combined Toxicity

Although the combined toxicity of MPs and copollutants is highly variable depending on exposure conditions and species‐specific differences, a number of relatively consistent molecular and cellular pathways have been identified across multiple studies. Oxidative stress is the most frequently reported mechanism, characterized by the overproduction of *ROS* and subsequent depletion of antioxidant defenses, leading to lipid peroxidation, protein damage, and DNA oxidation [31, 51, 52]. This oxidative imbalance often triggers inflammatory responses via the activation of NF‐κB and NLRP3 inflammasome pathways, leading to the release of proinflammatory cytokines [60, 94].

Endocrine disruption represents another major pathway, with MPs and their adsorbed pollutants interfering with hormone synthesis, transport, and signaling. For instance, coexposure to MPs and heavy metals or plastic additives has been shown to alter sex hormone levels and disrupt hypothalamic–pituitary–gonadal (HPG) axis function [59, 78, 96]. Additionally, mitochondrial dysfunction and apoptotic/necroptotic cell death pathways, including PANoptosis, have been documented in multiple organ systems following combined exposure [55, 60].

These pathways are often interconnected, and oxidative stress can activate inflammatory cascades, which in turn exacerbate endocrine disruption and cellular damage. Understanding these core mechanisms is crucial for predicting the health outcomes of combined toxicological effects associated with MPs and for developing targeted intervention strategies.

## 5. Perspectives and Conclusion

### 5.1. Current Research Limitations and Future Directions

The combined toxicity of MPs and multiple pollutants varies significantly among different species and their respective biological targets. Among numerous influencing factors, these can be categorized into MPs characteristics, coexposure pollutants, exposure factors, and environmental media characteristics. MPs‐related factors include the type [160], size [63], functional groups [161], charge [162], aging degree [163], and adsorption capacity [164]; coexposure pollutants factors include type, concentration, dissolved organic matter, and the impact of surfactants [161]; exposure factors include the target species, exposure time [165], exposure concentration [166], exposure scenarios [167], and polymer properties [168]. Environmental media factors can be further divided into water, soil, and air. In aquatic environments, factors include solution pH, salinity [169], octanol‐water partition coefficient [160], and hypoxia levels [170]; in soils, factors such as organic matter, enzyme activity, bacterial colonies [115], and physical–chemical properties [171] are relevant; in air, factors include temperature [172], precipitation, humidity, UV radiation, wind, and levels of airborne particulate matter [173]. Additionally, some studies have explored mitigating factors for joint toxicity, such as certain signaling pathways [174], inhibitors, antioxidants like N‐acetylcysteine (NAC) [94], humic acid [175], and plant growth‐promoting rhizobacteria [176]. Overall, the scope of research into the factors affecting the combined effects of MPs and other pollutants is expanding.

Current research still faces many significant challenges. For instance, the experimental exposure concentrations are often higher than actual environmental levels, and there is a lack of human biomonitoring data based on actual exposure scenarios. The molecular interaction mechanisms of combined toxicity have not been fully elucidated, particularly the interfacial interactions between nano‐sized plastic particles and biological contaminants like viruses, which urgently need further exploration. Moreover, existing assessment frameworks have yet to fully integrate the dynamic relationships between multimedium environmental behavior and bioaccumulation processes, making it difficult to reveal the cumulative toxicity and transgenerational effects of MPs and other pollutants under long‐term low‐dose combined exposure.

The standardization of exposure models and analytical methods is a critical priority. Current studies vary widely in MP types, sizes, concentrations, and exposure durations, limiting comparability and reproducibility [177, 178]. Harmonized exposure protocols should include environmentally relevant concentrations, characterized MP properties, and defined exposure routes and durations [179]. Analytical methods also require standardization. Techniques such as Fourier‐transform infrared spectroscopy (FTIR), Raman spectroscopy, and pyrolysis–gas chromatography–mass spectrometry (Py‐GC/MS) differ in sensitivity and applicability, leading to inconsistent data across laboratories [180]. Harmonized protocols for sample preparation and analysis are essential. International organization (GESAMP, ISO 16094‐2:2025) is developing standardized guidelines. Future research should align with these initiatives to improve data reliability and support evidence‐based policy development.

Future research should establish a multiscale assessment framework, integrating exposomics and metabolomics technologies to systematically analyze the dose–response relationship of combined exposure to MPs and multiple pollutants. Such efforts should be built upon standardized exposure protocols and analytical methods to ensure data comparability across studies. Furthermore, in vitro models based on organ‐on‐chip technology and high‐content imaging should be developed to facilitate mechanism studies across biological levels. A dynamic environmental–biological interface model should be constructed to quantify the regulation of pollutant bioavailability by MPs carrier effects. These breakthroughs will facilitate the development of mechanism‐based combined risk assessment models, providing theoretical support for establishing environmental benchmark and targeted mitigation strategies.

### 5.2. Conclusion

As emerging environmental vector, MPs can form a composite exposure system with multiple pollutants, including heavy metals, organic pollutants, plastic additives, antibiotics, and pathogens, through physical adsorption/desorption, chemical complexation, and biological membrane mediation. These combined exposures result in synergistic, additive, or antagonistic toxicity effects. The combined toxicity can span across multiple biological levels, including cells, tissues, organs, and individuals, manifesting as typical phenotypes such as enhanced oxidative stress, metabolic pathway abnormalities, immune dysfunction, and multigenerational developmental toxicity. Key parameters, including the size characteristics, surface properties, and environmental aging of MPs, significantly regulate these combined toxicity effects. Furthermore, endogenous antioxidant defense systems and detoxifying enzyme activities within organisms serve as important mechanisms for mitigating toxicity.

## Author Contributions

Resources, Lizhi Wu; writing–original draft preparation, Yuting Zhou; writing–review and editing, Mingluan Xing and Huixia Niu; visualization, Lizhi Wu and Xueqing Li; supervision, Mingluan Xing; project administration, Zhijian Chen and Xiaoming Lou; research design, Xiaoming Lou.

## Funding

This study was supported by the Noncommunicable Chronic Diseases‐National Science and Technology Major Project (Grant No. 2024ZD0531600); Zhejiang Science and Technology Plan for Disease Prevention and Control (Grant No. 2025JK158); Zhejiang Provincial Project for Medical Research and Health Sciences (Grant No. 2025KY758); Zhejiang Provincial Natural Science Foundation of China (LMS25H260006); Disease Prevention and Control Innovation Team of Zhejiang Province (2026JKC‐02).

## Disclosure

The statements, opinions and data contained in all publications are solely those of the individual author(s) and contributor(s) and not of the editor(s). The editor(s) disclaim responsibility for any injury to people or property resulting from any ideas, methods, instructions or products referred to in the content. All authors have read and agreed to the published version of the manuscript.

## Ethics Statement

The authors have nothing to report.

## Consent

The authors have nothing to report.

## Conflicts of Interest

The authors declare no conflicts of interest.

## Data Availability

The authors have nothing to report.
